# The dual carbon emission effects of digital economy: Evidence from China

**DOI:** 10.1016/j.heliyon.2025.e42554

**Published:** 2025-02-12

**Authors:** Yanze Wang, Zhi Su, Xuanye Cai, Jian Yu

**Affiliations:** a School of Statistics and Mathematics, Central University of Finance and Economics, Beijing, 102206, China; b School of Economics, Central University of Finance and Economics, Beijing, 102206, China

**Keywords:** Digital economy, Industrial digitization, Digital industrialization, Carbon emission, Carbon intensity, Carbon inequality

## Abstract

Nowadays, the digital economy has become a new engine of economic growth around the world. How to utilize the digital economy to achieve a balance between economic development and carbon emissions reduction is an imperative yet challenging endeavor for developing countries. In this paper, based on two perspectives of digital economy, the digital industrialization and industrial digitization, we use the industrial and city-level data of China from 2002 to 2017 to analyze the dual effects of digital economy on carbon emission, carbon intensity and carbon inequality. The results show that the two branches of the digital economy have different impacts on carbon emissions with varying influence mechanisms. The industrial digitization increases the carbon emissions of traditional industries but reduces their carbon intensity and carbon inequality by influencing energy intensity and net profit. While the digital industrialization increases urban carbon emissions and reduces urban carbon inequality through increasing energy intensity and optimizing industrial structure. Following the evidence from China, developing countries can allocate resources for digital industrialization or industrial digitization according to their own resource endowment and industrial status, thereby realizing the co-development of the economy and climate governance under limited resources.

## 1 Introduction

Reducing carbon emissions and carbon inequality are of paramount importance in the contemporary global environmental discourse (United Nations, 2021 [1]). The global carbon emissions have grown at an annual rate of 1.5 percent over the past decade with serious carbon inequality issues. This has led to severe social and environmental consequences, including the displacement of families, the destruction of homes, and the destabilization of communities [2,3]. Therefore, countries all around the world were compelled to devise multidimensional strategies to enhance energy conservation, reduce carbon emissions and minimize carbon inequality ([4]; United Nations, 2021 [5]). How to promote carbon reduction and realize the global climate governance goals has become the key research object of global society, especially for the developing countries with limited resources.

As a representative of developing countries, China has made remarkable achievements in economic development since the beginning of reform and opening up. However, the crude "high consumption, high pollution, low efficiency" development has both expanded the economy and caused great damage to the environment [6]. The substantial economic volume established upon the fossil energy consumption has resulted in extensive carbon emissions for China [7]. As the largest carbon emitter in the world, China is under tremendous pressure of taking immediate action to reduce carbon emissions [8]. In addition, China also faces the carbon inequality problem caused by the disparities in environmental policies, economic growth, industry, and population distribution [9].

In recent years, China has been committed to developing emerging high-tech industries such as the digital industry to achieve economic growth and environmental governance. The digital technology can leverage its efficient information processing capabilities to realize the digitization of traditional industries and the industrialization of digital technologies, thereby meeting a variety of e-needs in communications, transactions, and collaboration [10]. Based on digital technology, the digital economy provides consumers with a wide range of creative products and services, driving changes in production patterns and energy systems. As a result, its impact on carbon emissions has received extensive attention from the academic community.

Therefore, in this paper, we employ the panel regression model using the industrial and city-level data of China from 2002 to 2017 to analyze the influence of the digital economy on carbon emission, carbon intensity and carbon inequality. The empirical results indicate that the impact of the digital economy on carbon emissions is a multifaceted process. From the perspective of industrial digitization, by influencing energy intensity and net profit, the digital economy increases the carbon emissions of traditional industries, but also reduces their carbon intensity and carbon inequality. From the perspective of digital industrialization, through increasing energy intensity and optimizing industrial structure, the development of the digital economy increases urban carbon emissions and reduces urban carbon inequality.

This paper mainly contributes to the extant literature as follows: First, existing studies ignore the decomposition of the digital economy when examining its effects on carbon emissions. Therefore, we categorize the digital economy into digital industrialization and industrial digitization to conduct a comprehensive analysis of its impact on carbon emissions. Second, we discuss the impact of the digital economy on carbon emissions at the city and industry levels from the perspectives of total carbon emissions, carbon intensity, and carbon inequity. The results show that the industrial digitization effectively reduces both total carbon emissions and intensity, and reduces the level of carbon inequality, while digital industrialization increases total carbon emissions but likewise reduces the level of carbon inequality.

This paper is organized as follows: Section 2 provides a review of the existing literature. Section 3 introduces the theoretical model, the empirical models, and the data pre-processing procedure. Section 4 shows the empirical results of our research, and Section 5 summarizes our findings and presents relevant policy implications.

## 2 Literature review

The related literature covers three main aspects: the definition of the digital economy, the impact of the digital economy on the energy economy, and the impact of the digital economy on carbon emissions in different dimensions.

The first branch of literature introduces the definition of the digital economy. The concept of the digital economy was first proposed by Tapscott [11], which was initially conceived as electronic transactions mediated by information and communication technologies (ICT) [12,13]. The Internet has gradually been incorporated into the definition of the digital economy as its ability to facilitate commercial transactions has been recognized. Accordingly, the narrow definition of the digital economy emerged in 1999 when the U.S. Department of Commerce published “The Emerging Digital Economy”, which defined the digital economy as a mix of the e-commerce and the information technology industry. Subsequently, in conjunction with the research of Brynjolfsson & Kahin [14] and Lerner et al. [15], the conceptualization of the digital economy was further enriched to include digital infrastructure, digitized enterprises, electronic commerce, and computer networks. In recent years, there has been a growing international focus on the use of digital technologies, products, and services to facilitate industrialization. Several countries have subsequently revised their overview of the digital economy, emphasizing the importance of “digitization” in their definitions. In 2013, the Australian government defined the “digital economy” as the economic activities enabled by information technologies such as the Internet, mobile and sensor networks. In 2016, the “G20 Digital Economy Development and Cooperation Initiative” released during the G20 Hangzhou Summit defined the digital economy as “a series of economic activities that use digitized knowledge and information as key production factors, utilize modern information networks as important carriers, and leverage the effective use of postal and telecommunications information and communication technology as a crucial driving force for efficiency enhancement and economic structural optimization” [16]. In 2019, the National Statistics Office of the United Kingdom defined the digital economy as all economic activities that rely on digital technologies.

Till now, the digital economy can be divided into three levels: The first level is the basic concept, which focuses primarily on the digital (IT/ICT) domain including information services and telecommunications. The second level is the narrow definition of the digital economy, which includes e-business, digital services, and the platform economy, corresponding to the era of digital industrialization. The third level denotes the broad definition, often referred to as industrial digitization, which encompasses the digitization of almost all industries [17].

The second branch of literature primarily explores the impact of the digital economy on the energy economy. The digital economy has become a new catalyst for social expansion, with profound implications for economic development and environmental sustainability [18]. De Sousa Jabbour et al. [19] investigated the constrains between Industry 4.0 and environmentally sustainable manufacturing, discovering a significant positive correlation between the digital economy and energy intensity. Lange et al. [20], Santarius et al. [21], and Li et al. [18] demonstrated that the application of information and communication technologies (ICT) requires significant material and energy resources, thereby influencing the energy intensity of production. By analyzing the implementation of the “Broadband China” strategy, Li et al. [22] and Wang & Shao [23] revealed that the digital economy has a significant positive impact on energy intensity with obvious regional heterogeneity and resource endowment heterogeneity. Chen et al. [24] demonstrated that the digital economy may significantly contribute to the sustainability of environment. Shahbaz et al. [25], Ali et al. [26] and Li et al. [27] found that the digital economy can stimulate the renewable energy transition by improving the governance capacity of governments. In addition, Wan & Shi [28], Hu [29] and Guo et al. [30] indicated that the digital economy can effectively mitigate pollutant emissions such as PM2.5 and sulfur dioxide by stimulating technological innovation and energy efficiency improvement.

The third branch of literature discusses the impact of the digital economy on carbon emissions. Many scholars try to explore the relationship between digital economy development and carbon emissions reduction based on a variety of data and methods. Zhu et al. [31] empirically studied on the relationship between the digital economy and carbon emission intensity using data from 30 Chinese provinces between 2009 and 2019, and found that digital economy has a significant inhibitory effect on carbon emissions through the innovation effect and the upgrade of industrial structure. Hu [29] employed over a decade of city-level panel data and a dynamic difference-in-differences approach to investigate the dynamic changes in carbon emissions under the development of the digital economy, finding that digital economy has a sustainable carbon reduction effect. Similar conclusions were also reached by other influential works [26,30,32,33]. However, considering that a substantial portion of electricity is generated from non-renewable sources, the development of digital economy could lead to an increase in global electricity consumption and carbon emissions [34]. Specifically, Li & Wang (2022) [35] suggested an inverted U-shaped relationship between carbon emissions and the digital economy, which is attributed to short-term effects driven by increased energy consumption and progress in non-green technology, whereas long-term effects are driven by green technology improvements and industrial structure upgrades. Furthermore, in the pursuit of carbon peaking and carbon neutrality, a critical topic thorough exploration is ensuring fairness, equity, and efficiency in the emission reduction process [36,37]. Carbon inequality can be a reflection of disequilibrium in economic development and disparities of public welfare [38], and even an issue of development rights, interests and responsibilities [39]. Therefore, understanding the internal dynamics of carbon inequality across different regions and industries can significantly enhance our comprehension of carbon neutrality [9,40]. In light of this, some scholars have investigated carbon inequality and its driving factors, reaching the key conclusion that socio-economic elements are crucial for carbon inequality [41,42]. As the world’s largest carbon dioxide emitter, China is known for its serious carbon inequality [38]. Given that the digital economy is a powerful driver of socio-economic reforms at this stage, investigating its impact on carbon inequality is of great significance.

In conclusion, the existing literature provides a comprehensive account of the evolution of the conceptualization of the digital economy, reflecting the rapid advancements in digital technologies and their deepening integration into economic systems. Initially centered on electronic transactions, the scope of the digital economy has significantly broadened to include critical elements such as digital infrastructure, digital services, and the digitization of industries. Consequently, the digital economy has emerged as a pivotal driver of economic transformation, influencing a wide array of sectors and reshaping traditional economic paradigms. Moreover, scholars have extensively documented the profound and multifaceted impacts of the digital economy on energy economics and environmental sustainability. Notably, the digital economy plays a crucial role in affecting carbon emission, intensity and inequality, promoting renewable energy transitions, and mitigating pollutant emissions through technological innovation and efficiency improvements. These advancements underscore the potential of digitalization to contribute positively to global sustainability goals, particularly in the context of increasing environmental concerns.

However, despite these significant strides in understanding the interconnection between the digital economy and carbon emissions, the extant literature still presents several limitations. While the existing studies have effectively delineated multiple dimensions of the digital economy, most research has tended to aggregate these dimensions into a single, overarching digital economy development indicator. This approach often lacks the granularity necessary to explore the specific impacts of each dimension on carbon emissions, carbon intensity, and carbon equity. As a result, the heterogeneity of the impacts across different digital economy dimensions is frequently overlooked, limiting the precision and effectiveness of strategic interventions, particularly in the context of achieving China's carbon peaking and carbon neutrality goals. This calls for a more nuanced analysis that can inform targeted policy-making.

In contrast to previous studies, this paper adopts a more refined delineation methodology, going beyond modular analysis to examine in-depth structural and paradigmatic changes. It distinguishes between digital transformation within firms (industrial digitization) and technologically oriented structural changes in the broader economy (digital industrialization). This bidirectional approach not only highlights the distinction between internal and external drivers but also offers new insights into how the digital economy influences carbon emissions and carbon intensity through structural adjustments. Furthermore, this paper introduces a novel analytical perspective by incorporating the distributional effects of digital economic development, specifically focusing on carbon emission inequality. This approach is relatively rare in existing studies and sheds light on the social justice implications of digital economy. By addressing these equity concerns, the paper provides a theoretical foundation and empirical evidence for the development of more inclusive and equitable environmental policies. Moreover, this perspective enriches the discourse on the digital economy by integrating considerations of social fairness, thus contributing to a more holistic understanding of the digital economy’s influence on both economy and environment.

## 3 Theoretical analysis

### 3.1 The impact of industrial digitization on carbon emissions, intensity and inequality

1. Impacts on carbon emissions and pathways:

Industrial digitization has emerged as a significant driver of economic growth in contemporary society. It has introduced a new dynamism into economic activities, enhancing productivity and resource utilization efficiency. As digitalization progresses, its influence on carbon emissions evinces a multifaceted double effect. On the one hand, digitalization mitigates the intensity of carbon emissions to a certain extent [43,44]. On the other hand, alongside the expansion of economic activities, digitalization also precipitates an increase in the total amount of carbon emissions [null]; [45]. The mediating effect of enterprise net profit represents a pivotal pathway, industrial digitization has the effect of enhancing the operational efficiency and competitiveness of firms, which in turn affects their profitability [46,47]. The net profit of an enterprise may serve as an indicator of the economic benefits derived from the digitization process. Nevertheless, the impact of elevated carbon emissions resulting from substantial expansion in enterprise economic activity cannot be disregarded [48]. In light of the aforementioned theoretical analysis, this paper puts forth the following hypotheses.

Hypothesis 1 Industrial digitization increases carbon emissions.

2. Impacts on carbon intensity and pathways:

The impact of industrial digitization on carbon intensity is significant and multifaceted. While initial increases in energy consumption due to the integration of new technologies are evident, subsequent improvements in efficiency ultimately lead to a reduction in carbon intensity. In the Chinese logistics and manufacturing sectors, studies have demonstrated that the implementation of digital technologies can result in a reduction of carbon intensity through the enhancement of energy efficiency and the promotion of the adoption of green technologies [49]. In this context, the mediating role of energy consumption intensity is of particular significance. Digitalization facilitates more efficient resource allocation and curbs wasteful energy consumption, which in turn precipitates a marked decline in carbon intensity over time [50]. Moreover, enterprise net profit plays a pivotal mediating role in this process. Net profit can serve as a reflection of the economic benefits derived by enterprises throughout the digitalization process. Changes in such benefits act as a mediator in the impact of digital transformation on carbon emission intensity [51,52]. In light of the aforementioned theoretical analysis, this paper puts forth the following hypotheses.

Hypothesis 2 Industrial digitization reduces carbon intensity.

3. Impacts on carbon inequality and pathways:

Industrial digitization has played an instrumental role in the reduction of carbon inequality. The advancement of industrial digitization not only fosters the development of green technology but also reduces energy consumption intensity, particularly in the central and western regions of China. This has the effect of narrowing the carbon emission gap between regions [53]. Moreover, industrial digitization has been demonstrated to have a substantial impact on carbon reduction in high-polluting industries, thereby contributing to a reduction in carbon inequality between industries [43]. In conclusion, industrial digitization has been demonstrated to significantly reduce carbon inequalities at both the regional and industrial levels, which is achieved by promoting productivity, energy efficiency and green innovation [54]. In light of the aforementioned theoretical analysis, this paper puts forth the following hypotheses.

Hypothesis 3 Industrial digitization diminishes carbon inequality.

### 3.2 The impact of digital industrialization on carbon emissions, intensity and inequality

1. Impacts on carbon emission and pathways:

As an emerging field, digital industrialization plays a pivotal role in shaping the future of the digital economy. It serves as the backbone for various digital advancements, facilitating the integration of cutting-edge technologies such as artificial intelligence (AI), automation, Internet of Things (IoT), and cloud computing into traditional industrial processes. However, the environmental implications of digital industrialization, particularly concerning carbon emissions, are highly complex and multifaceted. At present, digital industrialization has a considerable impact on total carbon emissions. The introduction of sophisticated digital technologies, including AI, automation, and cloud computing, frequently leads to a substantial increase in electricity consumption. These technologies, while improving operational efficiency, require extensive computational power and data storage capabilities, which in turn place significant demands on energy resources [55,56]. For instance, data centers that support cloud computing and AI workloads are known to be energy-intensive, with some studies indicating that their energy use could rival that of small nations [57]. In light of the aforementioned theoretical analysis, this paper puts forth the following hypothesis.

Hypothesis 4 digital industrialization increases carbon emissions.

2. Impacts on carbon intensity and pathways:

The impact of digital industrialization on carbon intensity is mediated by the intensity of electricity energy consumption and industrial structure. The initial impact of digital industrialization on energy consumption is an increase due to the deployment of energy-intensive digital technologies [58]. Concurrently, industrial structure serves as a mediating factor, enabling transitions to sectors with lower energy intensities. As digital technologies evolve, industries are presented with the opportunity to transition from heavy, energy-dependent sectors such as manufacturing to lighter, technology-driven sectors such as services. This structural shift results in a reduction in the amount of energy required per unit of GDP, thereby reducing carbon intensity [59,60]. In light of the aforementioned theoretical analysis, this paper puts forth the following hypotheses.

Hypothesis 5 digital industrialization increases carbon intensity.

3. Impacts on carbon inequality and pathways:

Digital industrialization plays a significant role in addressing carbon inequality by reshaping industrial structures and reducing disparities in carbon emission reductions across different industries. Specifically, digital industrialization drives the shift of traditional energy-intensive sectors, such as manufacturing and heavy industry, toward more advanced, technology-driven industries such as information technology and services. As these structural changes unfold, the overall reduction in carbon emissions becomes evident, thereby contributing to the convergence of carbon emission levels across industries and diminishing the disparity in carbon emission between different sectors [43]. In light of the aforementioned theoretical analysis, this paper puts forth the following hypotheses.

Hypothesis 6 digital industrialization reduces carbon inequality.

## 4 Methodology and data pre-processing

### 4.1 STIRPAT model

This paper is based on the STIRPAT (stochastic impacts by regression on population, affluence, and technology) model proposed by Rosa and Dietz [61]. The STIRPAT framework was developed upon the standard IPAT model [62], which briefly illustrates the influence of population (P), affluence (A), and technology (T) on environment (I). The general mathematical expression of the STIRPAT model is provided as follows:

(1) $$I=aP^{b}A^{c}T^{d}\varepsilon$$

Where P denotes population size, a represents affluence, and T represents technological level. a is a constant, and b, c, and d are the exponential control parameters for population, affluence, and technological level, respectively. ε represents the random error term. To reflect the differing proportional impacts of various factors on the environment and eliminate potential heteroscedasticity, the STIRPAT model is logarithmically transformed as:

(2) LnI=a+b(LnP)+c(LnA)+d(LnT)+Lnε

### 4.2 Mediation effect model

According to the mediation effect model proposed by Baron & Kenny [63], this paper adopts the three-step method to test the influence path of the digital economy on carbon emission, carbon intensity and carbon inequality as follows:

(3) $$Y=cX+u_{1}$$

(4) $$M=aX+u_{2}$$

(5) $$Y=c^{\prime }X+bM+u_{3}$$

Equation (3) tests the effect of the core explanatory variable X on the explained variable Y. Subsequent analysis can proceed if the effect is statistically significant. Equation (4) tests the relationship between X and the mediation variable M. Equation (5) explores how X impacts Y through M. If the effect of M on Y is significant, there are two possible scenarios: A statistically significant coefficient $c^{\prime }$ suggests that X affects Y through both direct and mediated paths, indicating a partial mediation effect. On the contrary, a lack of significance in $c^{\prime }$ indicates that the effect of X on Y is entirely mediated through M, establishing a complete mediation effect.

### 4.3 Two-way fixed effects (TWFE) model

We rely on a panel regression model to quantify the relationship between digital economy and carbon emissions. Specifically, this paper divides the digital economy into two perspectives: digital industrialization and industrial digitization. For meticulous consideration, multiple proxy variables are selected to represent the digital industrialization, industrial digitization, carbon emission, carbon intensity and carbon inequality.

Regarding the independent variables, existing literature predominantly focused on developing digital economy indicator systems and indices to assess the composite impact of the digital economy at the provincial and city levels in China ([64]; Li et al., 2021; Pan et al., 2022 [65]). However, these methodologies share the common perspective of examining the digital economy holistically as a unified entity. Therefore, we build an index system in accordance with the research theme, based on the industrial digitization and digital industrialization dimensions. In contrast to the existing literature, this approach ensures the analysis of the different development trajectories and dimensions of the digital economy. The postal and telecommunications service expenditures, the postal and telecommunications services revenue, and the ratio of postal and telecommunications revenue to GDP are selected as the proxy variables for dual perspectives. Firstly, based on the research of Chen et al. (2023) [66], Song et.al [67] and Guo et al. (2023), the level of postal and telecommunications revenue and expenditure is identified as an important and common constituent for digital economy indices. Secondly, to attenuate confounding interference from different variables and enhance comparability between two separate dimensions, the level of postal and telecommunications revenue and expenditure appear as appropriate unified proxy variables that allow for consonance between two datasets.

#### 4.3.1 Industrial digitization

In the industrial digitization part, we construct the Equations (6), (7), (8), (9) as regression models.

(6) $$\ln\;{\;carbon}_{it}=\beta_{0}+\beta_{1}\;\ln\;{DE}_{it}+\beta \;control+city_{i}+year_{t}+\varepsilon_{it}$$

(7) $$\ln\;{\;emmi}_{it}=\beta_{0}+\beta_{1}\;\ln\;{DE}_{it}+\beta \;control+city_{i}+year_{t}+\varepsilon_{it}$$

(8) $$\ln\;{\;emmiten}_{it}=\beta_{0}+\beta_{1}\;\ln\;{DE}_{it}+\beta \;control+city_{i}+year_{t}+\varepsilon_{it}$$

(9) $${gini}_{it}=\beta_{0}+\beta_{1}\;\ln\;{DE}_{it}+\beta \;control+city_{i}+year_{t}+\varepsilon_{it}$$

In this section, we use the expenditure on postal and telecommunications service as the proxy variable of Digital Economy (DE). Following Chen et al. [68], Guo et al. [69] and Khanna & Sharma [70], we select the total corporate assets, fossil energy consumption, debt-to-asset ratio, net profit, and independent R&D investment as the control variables to reflect the industrial information of population (P), affluence (A) and technological level (T). This allows us to isolate the influence of population, economic growth and technological progress on carbon emissions, carbon intensity and carbon inequality. The specific descriptions of variables are given in Table 1 as follows:

**Table 1:** Variables description of industrial digitization.

Variables	Description	Unit
*carbon*	Total carbon emissions	Thousands of tons
*emmi*	Carbon intensity (divided by operating revenue)	Tons per thousand yuan
*eminten*	Carbon intensity (divided by total output)	Tons per thousand yuan
*gini*	Carbon inequality Gini index	— —
*postal_telecom_expen*	Expenditures of postal and telecommunications service	Thousands of yuan
*total_asset*	Total corporate assets	Millions of yuan
*fuel_consum*	Fossil energy consumption	Thousands of tons
*debt_ratio*	Debt-to-asset ratio	%
*inner_inno*	Independent R&D Investment	Thousands of yuan
*energy_y*	Energy consumption intensity	Tons per thousand yuan
*net_profit*	Corporate net profits	Thousands of yuan

#### 4.3.2 Digital industrialization

For the digital industrialization part, we construct the Equations 10 to 15 as regression models.

(10) $$\ln\;{\;carbon}_{jt}=\beta_{0}+\beta_{1}\;\ln\;{DE1}_{jt}+\beta \;control+{prov}_{j}+year_{t}+\varepsilon_{jt}$$

(11) $$\ln\;{\;car\_inten}_{jt}=\beta_{0}+\beta_{1}{lnDE1}_{jt}+\beta \;control+{prov}_{j}+year_{t}+\varepsilon_{jt}$$

(12) $${\;gini}_{jt}=\beta_{0}+\beta_{1}\;\ln\;{DE1}_{jt}+\beta \;control+{prov}_{j}+year_{t}+\varepsilon_{jt}$$

In this section, we use the postal and telecommunications services revenue as the proxy variable of Digital Economy (DE). In term of control variables, analogous to the digital industrialization framework, population (P), affluence (A), and technology (T) are selected. Specifically, city population size and regional gross domestic product per capita are directly employed as proxy variables for population and affluence respectively. Concerning technological level, following Lin & Zhu [71] and Khanna & Sharma [70], this paper designates R&D expenditure as a proxy variable. The specific descriptions of variables are given in Table 2 as follows:

**Table 2:** Variables description of digital industrialization.

Variables	Description	Unit
*carbon*	Total carbon emissions	Tons
*car_inten*	Carbon intensity (divided by fiscal expenditure)	Tons per yuan
*gini*	Carbon inequality Gini index	— —
*postal_telecom_revenue*	Postal and telecommunications services revenue	Millions of yuan
*gdp_per_capita*	Regional gross domestic product per capita	Thousands of yuan
*pop*	City population size	Tens of thousands of people
*science*	R&D expenditure	Millions of yuan
*industry_stru*	Industrial structure	%
*energy_inten*	Electricity energy consumption intensity	kWh per yuan

### 4.4 Data pre-processing

The dependent variables of this paper include carbon emission, carbon intensity, and carbon inequality. The carbon emission is measured using the fossil fuel consumption data from the China City Statistical Yearbook, the China City Energy Statistical Yearbook and the Chinese National Tax Survey Database. The carbon intensity is measured by dividing carbon emissions data by cities’ fiscal expenditures. The carbon inequality is measured by Gini index, which is calculated with the data of per capita carbon emissions of counties and the per capita carbon emissions of corporations. According to Sen [72], the Gini index (G) is calculated with the Equation 16 as follows:

(16) $$G=\frac{\sum_{i=1}^{n}\sum_{j=1}^{n}\left|x_{i}-x_{j}\right|}{2n^{2}\bar{x}}\text{,}$$

where $x_{i}$ is the carbon emissions of i, $\bar{x}$ is the average value of x, n is the number of samples.

Specifically, the data of industrial digitization contain 82 Chinese industries from 2009 to 2015 of 396 cities, which was collected from the Chinese National Tax Survey Database. To eliminate the influence of outliers, we excluded samples without essential variable data and subjected all three dependent variables to a 1 % winsorization procedure. The descriptive statistics of the data are given in Table 3.

**Table 3:** Descriptive statistics of industrial digitization.

Variables	Obs.	Mean	SD	Min	Max
*postal_telecom_expen*	34110	174.19	8208.07	0.02	1411373
*carbon*	34110	91871.21	3959291.46	0.00	671266000
*eminten*	34110	72.36	8513.62	0.00	1307253
*emmi*	34110	19.95	2702.69	0.00	490619
*gini*	22126^a^	0.38	0.31	0.00	0.99
*debt_ratio*	32773	0.56	0.22	0.00	1.00
*inner_inno*	34110	823.15	11579.83	−4.52	1216681
*total_asset*	34110	345447.25	3049296.91	−507.52	248672180
*fuel_consum*	34110	21449.75	1480731.44	0.00	262152780
*energy_y*	34110	21.78	2800.40	0.00	489647
*net_profit*	34093	9953.34	186626.69	−12593593	14218893

a The Gini index has a relatively small sample size due to the data matching considerations and the requirement to have at least two sub-sample in each city or industry.

The data of digital industrialization contain 301 cities from 2002 to 2017, which were obtained from the China City Statistical Yearbook and the China City Energy Statistical Yearbook. To eliminate the influence of outliers, we excluded the samples with missing values from the regression. The descriptive statistics of the data are given in Table 4.

**Table 4:** Descriptive statistics of digital industrialization.

Variables	Obs.	Mean	SD	Min	Max
*postal_telecom_revenue*	4423	341062.53	694640.15	4679	13964015
*carbon*	4423	23.99	22.67	1.37	230.71
*car_inten*	4423	0.002	0.002	0.000	0.012
*gini*	3984	0.19	0.11	0.00	0.71
*gdp_per_capita*	4423	32216.21	28651.31	99.00	467749
*pop*	4422	438.20	389.99	15.97	11098.40
*science*	4151	50653.20	208792.48	0.00	3898971
*energy_inten*	4339	0.07	0.09	0.00	2.05
*industry_stru*	4201	0.84	0.43	0.09	9.48

## 5 Benchmark results

### 5.1 Industrial digitization

To enhance the robustness of empirical results, we construct two fixed effects models to examine the impact of industrial digitization on carbon emissions, carbon intensity, and carbon inequality, as shown in Table 5. The control variables include the features of population, affluence, and technological level. We also control the time and city fixed effects to mitigate the confounding effects of time-variant and city-level shocks. Column (1) presents a significantly positive correlation between postal and telecommunications service expenditures and total carbon emissions at the 1 % significance level. Specifically, a 1 % increase in digital expenditure leads to a 0.142 % increase in total carbon emissions. This suggests that the direct energy consumption and indirect effects (e.g. equipment manufacturing and waste disposal) of industrial digitization contribute to the increase in total carbon emissions, despite its efficiency and energy-saving potential. Columns (2) and (3) show that the impacts of postal and telecommunications service expenditures on dual carbon emission intensity are significantly negative at the 1 % significance level. Specifically, a 1 % increase in postal and telecommunications service expenditures leads to a decrease of 0.011 % in carbon emission intensity divided by operating revenue and a decrease of 0.012 % in carbon emission intensity divided by total output. This reduction could be driven by the application of industrial digitization, which enhances production efficiency and promotes the optimal resources allocation, thereby reducing the energy consumption and carbon emissions per unit of product or service. Column (4) shows that the impact of postal and telecommunications service expenditures on the Gini index is significantly negative at the 1 % significance level. Specifically, a 1 % increase in such expenditures leads to a 0.041 decrease in Gini index. The industrial digitalization is driving an economic structural transformation, which leads to a more equitable distribution of carbon emission reductions across different societal levels.

**Table 5:** The baseline regression results.

	(1)	(2)	(3)	(4)
	*log_carbon*	*log_emmi*	*log_eminten*	*gini*
*ln_postal_telecom_expen*	0.141∗∗∗	−0.015∗∗∗	−0.018∗∗∗	−0.024∗∗∗
	(0.007)	(0.002)	(0.002)	(0.002)
*ln_total_asset*	0.164∗∗∗	−0.027∗∗∗	−0.027∗∗∗	0.033∗∗∗
	(0.008)	(0.002)	(0.002)	(0.002)
*ln_fuel_consum*	0.345∗∗∗	0.046∗∗∗	0.050∗∗∗	0.000
	(0.004)	(0.001)	(0.001)	(0.001)
*debt_ratio*	0.375∗∗∗	−0.027∗∗	−0.038∗∗∗	−0.070∗∗∗
	(0.048)	(0.011)	(0.013)	(0.010)
*ln_inner_inno*	−0.061∗∗∗	−0.007∗∗∗	−0.007∗∗∗	0.022∗∗∗
	(0.004)	(0.001)	(0.001)	(0.001)
*cons*	2.097∗∗∗	0.318∗∗∗	0.350∗∗∗	0.133∗∗∗
	(0.072)	(0.017)	(0.019)	(0.015)
*N*	25787	25787	25787	18206
*R*^*2*^	0.628	0.169	0.161	0.253
*Industry_FE*	Yes	Yes	Yes	Yes
*Year_FE*	Yes	Yes	Yes	Yes

Standard errors in parentheses, ∗*p* < 0.1, ∗∗*p* < 0.05, ∗∗∗*p* < 0.01.

Regarding control variables, our findings indicate that total corporate assets, independent R&D investment, and fossil energy consumption exert significantly positive effect on four dependent variables. However, the effect of debt-to-asset ratio is only significant with respect to total carbon emissions.

### 5.2 Digital industrialization

To enhance the robustness of empirical results, we construct two fixed effects models to examine the impacts of postal and telecommunications services revenue (proxy variable for digital industrialization) on total carbon emissions, carbon intensity, and carbon inequality as shown in Table 6. The control variables include regional gross domestic product per capita, city population size and R&D expenditure. Year and province fixed effects are also incorporated to mitigate potential endogeneity issues arising from omitting variables related to time and province characteristics.

**Table 6:** The baseline regression results.

	(1)	(2)	(3)
	*ln_carbon*	*lncar_inten*	*gini*
*lnpostal_telecom_ revenue*	0.126∗∗∗	−0.014	−0.029∗∗∗
	(0.009)	(0.010)	(0.003)
*lngdp_per_capita*	0.426∗∗∗	0.234∗∗∗	0.000
	(0.014)	(0.016)	(0.005)
*lnpop*	0.526∗∗∗	0.075∗∗∗	0.026∗∗∗
	(0.013)	(0.015)	(0.005)
*lnscience*	0.101∗∗∗	−0.137∗∗∗	0.018∗∗∗
	(0.008)	(0.008)	(0.003)
*cons*	−6.943∗∗∗	3.607∗∗∗	0.218∗∗∗
	(0.136)	(0.152)	(0.049)
*N*	4149	4149	3738
*R*^*2*^	0.870	0.800	0.209
*Prov_FE*	Yes	Yes	Yes
*Year_FE*	Yes	Yes	Yes

Standard errors in parentheses, ∗*p* < 0.1, ∗∗*p* < 0.05, ∗∗∗*p* < 0.01.

Table 6 presents the effects of postal and telecommunications services revenue on carbon emission, carbon emission intensity and carbon inequality. Column (1) presents significantly positive correlation between core explanatory variable and total carbon emissions at the 1 % significance level. Specifically, a 1 % increase in explanatory variable leads to a 0.126 % increase in total carbon emissions. This suggests that, increased revenue from postal and telecommunications services, which are important components of the information and logistics infrastructure, often implies increased demand for services. As the demand for these services increases, there is a corresponding increase in logistics activities, data center operations and the use of communications equipment, which is often accompanied by an increase in energy consumption and, consequently, an increase in carbon emissions. This point highlights the link between growth in economic activity and environmental pressures. Column (3) presents that the impact of the explanatory variable on Gini index, is significantly negative at the 1 % significance level. Specifically, when the explanatory variable increases by 1 %, Gini index could decrease by 0.029. The result implies that the expansion of postal and telecommunications services play a pivotal role in mitigating socioeconomic inequalities. This could be attributed to the fact that these services provide a large number of employment opportunities and promote the flow of information, increasing market transparency and enhancing productive efficiency. However, there is no significance between two explanatory variables and carbon intensity.

### 5.3 Summary and comparison

The results of the benchmark regression analysis indicate that both digital industrialization and industrial digitization have a significantly positive impact on total carbon emissions. The advancement of digital industrialization not only contributes to the growth of total carbon emissions but also exerts a beneficial influence on the reduction of carbon emission inequality. Nevertheless, its impact on carbon emission intensity remains insignificant. While industrial digitization has a significant impact on reducing carbon emission intensity while simultaneously increasing total carbon emissions. This indicates that industrial digitization plays an important role in enhancing production efficiency and resource optimization. Furthermore, industrial digitization exerts a more pronounced impact on reducing carbon emission inequality, thereby facilitating a more equitable distribution of carbon emission.

The above findings are similar to those of previous studies on the impact of the digital economy in terms of total carbon emissions and intensity. Jing et al. [54] found that the digital economy contributes to the reduction of urban carbon intensity by promoting productivity and green innovation. However, the development of the digital economy can lead to an increase in energy consumption in specific regions, which may subsequently exacerbate the total carbon emissions. The study of Lyu et al. [73,74] has shown that while the digital economy reduces carbon intensity to some extent, it can lead to an increase in total carbon emissions, especially in the early stages of economic development. Zhong et al. [75] found that the digital economy has significantly reduced the intensity of agricultural carbon emissions by promoting technological advances in agriculture, but total carbon emissions remain at a high level. The results of these studies consistently demonstrate that the development of the digital economy typically results in an increase in total carbon emissions, while carbon intensity is simultaneously reduced. Other influential studies have yielded analogous findings [51,75]. Nevertheless, a limited corpus of literature indicates that, in certain instances, the advent of the digital economy may potentially contribute to a reduction in overall carbon emissions [29,31]. In regard to carbon inequality, the findings of this study indicate that both industrial digitization and digital industrialization exert a diminishing influence on carbon inequality. This result aligns with the findings of previous research in this field. For instance, the findings of Yang et al. [76] indicate that the unequal matching of allocations and targets in carbon abatement practices gives rise to abatement inequality. Conversely, the digital economy has been demonstrated to significantly reduce income inequality and the level of spatial clustering of the digital economy, while also enhancing the inclusiveness of digital finance. This, in turn, serves to reduce carbon inequality [77]. The digital economy serves as an illustrative case of carbon inequality. By evaluating the impacts of the digital economy over 2000–2018 and assessing the extent and manner in which the carbon costs and economic benefits embodied in the digital economy are unevenly distributed across regions globally, it becomes evident that carbon inequality decreases dramatically with the proliferation of trade in the digital economy.

## 6 Robustness analysis

### 6.1 Industrial digitization

#### 6.1.1 IV tests

To address the endogeneity concerns inherent in the benchmark regression, this study follows the approach conducted by Yi & Zhou [78], Wen et al. [79], and Yi et al. [80], by employing the one-period lagged expenditure on postal and telecommunications services as an instrumental variable (IV) and two-stage least squares (2SLS) regression for industrial digitization. The underlying justification for this selection is that the one-period lagged level of expenditure on postal and telecommunications services is typically highly correlated with the current level of industrial digitization, making it a strong predictor of the endogenous variable. Secondly, the temporal precedence ensures that the one-period lagged expenditure on postal and telecommunications services is not contemporaneously affected by carbon emission changes. It impacts carbon emissions only indirectly through its effect on industrial digitization, thereby satisfying the exogeneity condition of a valid instrument.

The empirical findings of the Instrumental variable regression are presented in Table 7. The coefficients for the one-period lagged level of expenditure on postal and telecommunications service are significant at the 1 % level, corroborating our baseline regression insight that industrial digitization significantly reduces carbon intensity and carbon inequality, while also increasing total carbon emissions.

**Table 7:** Instrumental variable test results.

	(1)	(2)	(3)	(4)
	*log_carbon*	*log_emmi*	*log_eminten*	*gini*
*L1_ln_postal_telecom_expen*	0.153∗∗∗	−0.012∗∗∗	−0.014∗∗∗	−0.035∗∗∗
	(0.008)	(0.001)	(0.002)	(0.002)
*ln_total_asset*	0.174∗∗∗	−0.019∗∗∗	−0.016∗∗∗	0.039∗∗∗
	(0.009)	(0.002)	(0.002)	(0.002)
*ln_fuel_consum*	0.319∗∗∗	0.037∗∗∗	0.040∗∗∗	0.002
	(0.005)	(0.001)	(0.001)	(0.001)
*debt_ratio*	0.526∗∗∗	−0.021∗	−0.029∗∗	−0.085∗∗∗
	(0.059)	(0.011)	(0.013)	(0.013)
*ln_inner_inno*	−0.054∗∗∗	−0.006∗∗∗	−0.006∗∗∗	0.021∗∗∗
	(0.004)	(0.001)	(0.001)	(0.001)
*N*	17003	17003	17003	10595
*R*^*2*^	0.361	0.097	0.086	0.103
*Industry_FE*	Yes	Yes	Yes	Yes
*Year_FE*	Yes	Yes	Yes	Yes

Standard errors in parentheses, ∗*p* < 0.1, ∗∗*p* < 0.05, ∗∗∗*p* < 0.01.

#### 6.1.2 Cluster standard error tests

To further evaluate the robustness of the results, this paper incorporates a standard error test for clustering at the city level. As shown in Table 8, the results of the adjusted regression analysis indicate that the influence of postal and telecommunication service expenditures on carbon emissions, carbon intensity, and carbon inequality remains significant. This suggests that the role of industrial digitization in these areas remains robust after controlling for intra-city autocorrelation. This finding further validates the reliability of the baseline conclusions, indicating that postal and telecommunication service expenditures, as a proxy variable for industrial digitization, exert a significant impact on carbon emission-related indicators.

**Table 8:** Cluster standard error regression results.

	(1)	(2)	(3)	(4)
	*log_carbon*	*log_emmi*	*log_eminten*	*gini*
*ln_postal_telecom_expen*	0.141∗∗∗	−0.015∗∗∗	−0.018∗∗∗	−0.024∗∗∗
	(0.013)	(0.002)	(0.002)	(0.003)
*ln_total_asset*	0.164∗∗∗	−0.027∗∗∗	−0.027∗∗∗	0.033∗∗∗
	(0.013)	(0.002)	(0.003)	(0.003)
*ln_fuel_consum*	0.345∗∗∗	0.046∗∗∗	0.050∗∗∗	0.000
	(0.007)	(0.002)	(0.002)	(0.001)
*debt_ratio*	0.375∗∗∗	−0.027∗	−0.038∗∗	−0.070∗∗∗
	(0.068)	(0.014)	(0.016)	(0.015)
*ln_inner_inno*	−0.061∗∗∗	−0.007∗∗∗	−0.007∗∗∗	0.022∗∗∗
	(0.010)	(0.001)	(0.001)	(0.002)
*cons*	2.097∗∗∗	0.318∗∗∗	0.350∗∗∗	0.133∗∗∗
	(0.118)	(0.023)	(0.031)	(0.026)
*N*	25787	25787	25787	18206
*R*^*2*^	0.628	0.169	0.161	0.253
*Industry_FE*	Yes	Yes	Yes	Yes
*Year_FE*	Yes	Yes	Yes	Yes

Standard errors in parentheses, ∗*p* < 0.1, ∗∗*p* < 0.05, ∗∗∗*p* < 0.01.

#### 6.1.3 Balanced panel regression

To further validate the robustness of the findings, this study conducts a balanced panel regression analysis and controls for industry and year fixed effects. As demonstrated in Table 9, the findings of the analysis indicate that the impacts of expenditure on postal and telecommunications services on carbon emissions, carbon intensity, and carbon inequality remain significant. The robustness test results provide further support the findings of the benchmark regression.

**Table 9:** Balanced panel regression results.

	(1)	(2)	(3)	(4)
	*log_carbon*	*log_emmi*	*log_eminten*	*gini*
*ln_postal_telecom_expen*	0.120∗∗∗	−0.008∗∗∗	−0.010∗∗∗	−0.017∗∗∗
	(0.016)	(0.002)	(0.003)	(0.004)
*ln_total_asset*	0.173∗∗∗	−0.021∗∗∗	−0.023∗∗∗	0.011∗∗
	(0.020)	(0.003)	(0.003)	(0.004)
*ln_fuel_consum*	0.194∗∗∗	0.019∗∗∗	0.020∗∗∗	0.014∗∗∗
	(0.010)	(0.001)	(0.002)	(0.002)
*debt_ratio*	0.850∗∗∗	−0.064∗∗∗	−0.078∗∗∗	−0.074∗∗
	(0.147)	(0.020)	(0.025)	(0.031)
*ln_inner_inno*	−0.032∗∗∗	−0.004∗∗∗	−0.005∗∗∗	0.011∗∗∗
	(0.007)	(0.001)	(0.001)	(0.002)
*cons*	2.412∗∗∗	0.327∗∗∗	0.380∗∗∗	0.446∗∗∗
	(0.205)	(0.028)	(0.035)	(0.045)
*N*	3566	3566	3566	2563
*R*^*2*^	0.655	0.169	0.182	0.259
*Industry_FE*	Yes	Yes	Yes	Yes
*Year_FE*	Yes	Yes	Yes	Yes

Standard errors in parentheses, ∗*p* < 0.1, ∗∗*p* < 0.05, ∗∗∗*p* < 0.01.

#### 6.1.4 High-dimensional fixed effects model

To further ensure the robustness of the baseline results, this study employs high-dimensional fixed effects regression (HDFER), which specifically includes industry-year high-dimensional fixed effects. The incorporation of industry and year interaction fixed effects enables the model to account for the unobservable inherent to each industry in distinct years, thereby enhancing its capacity to address potential heterogeneity and omitted variable issues.

Following the introduction of industry-year high-dimensional fixed effects, the results are presented in Table 10. These findings indicate that the impact of expenditure of postal and telecommunications services on total carbon emissions, carbon intensity, and carbon inequality remain significant. The robustness test results provide further support the findings of the benchmark regression.

**Table 10:** High-dimensional fixed effects regression results.

	(1)	(2)	(3)	(4)
	*log_carbon*	*log_emmi*	*log_eminten*	*gini*
*ln_postal_telecom_expen*	0.127∗∗∗	−0.011∗∗∗	−0.012∗∗∗	−0.025∗∗∗
	(0.007)	(0.002)	(0.002)	(0.001)
*ln_total_asset*	0.200∗∗∗	−0.025∗∗∗	−0.024∗∗∗	0.016∗∗∗
	(0.008)	(0.002)	(0.002)	(0.002)
*ln_fuel_consum*	0.322∗∗∗	0.043∗∗∗	0.046∗∗∗	0.008∗∗∗
	(0.004)	(0.001)	(0.001)	(0.001)
*debt_ratio*	0.203∗∗∗	−0.019	−0.029∗∗	−0.033∗∗∗
	(0.047)	(0.012)	(0.013)	(0.009)
*ln_inner_inno*	−0.022∗∗∗	−0.004∗∗∗	−0.005∗∗∗	0.009∗∗∗
	(0.004)	(0.001)	(0.001)	(0.001)
*cons*	1.885∗∗∗	0.286∗∗∗	0.307∗∗∗	0.284∗∗∗
	(0.073)	(0.018)	(0.020)	(0.014)
*N*	25569	25569	25569	18030
*R*^*2*^	0.719	0.286	0.283	0.508
*Industry_FE*	Yes	Yes	Yes	Yes
*Year_FE*	Yes	Yes	Yes	Yes
*City_Year_FE*	Yes	Yes	Yes	Yes

Standard errors in parentheses, ∗*p* < 0.1, ∗∗*p* < 0.05, ∗∗∗*p* < 0.01.

### 6.2 Digital industrialization

#### 6.2.1 IV tests

To address the endogeneity concerns inherent in the benchmark regression, this study follows the approach conducted by Yi & Zhou [78], Wen et al. [79], and Yi et al. [80], by employing the one-period lagged postal and telecommunications services revenue as an instrumental variable (IV) and two-stage least squares (2SLS) regression for digital industrialization. The underlying justification for this selection is that the one-period lagged level of postal and telecommunications services revenue is typically highly correlated with the current level of digital industrialization, making it a strong predictor of the endogenous variable. Secondly, the temporal precedence ensures that the one-period lagged postal and telecommunications services revenue is not contemporaneously affected by carbon emission changes. It impacts carbon emissions only indirectly through its effect on digital industrialization, thereby satisfying the exogeneity condition of a valid instrument.

The empirical findings of the instrumental variable regression are presented in Table 11. The coefficients of the one-period lagged level of postal and telecommunications services revenue with carbon emissions and carbon inequality are significant at the 1 % level and that with carbon intensity is significant at the 5 % level. In summary, through the IV regression, this study ascertains the consistency and robustness of our baseline finding that digital industrialization significantly increases carbon emissions and diminishes carbon inequality. It is noteworthy that, subsequent to the resolution of the endogenous issue, digital industrialization has been observed to exert a considerable negative influence on carbon intensity, a phenomenon that is not reflected in the baseline regression.

**Table 11:** Instrumental variable test results.

	(1)	(2)	(3)
	*lncarbon*	*lnemission_inten*	*gini*
*lnpostal_telecom_revenue*	0.158∗∗∗	−0.034∗∗	−0.041∗∗∗
	(0.013)	(0.014)	(0.005)
*lngdp_per_capita*	0.402∗∗∗	0.245∗∗∗	0.009
	(0.015)	(0.017)	(0.006)
*lnpop*	0.497∗∗∗	0.090∗∗∗	0.036∗∗∗
	(0.015)	(0.017)	(0.006)
*lnscience*	0.097∗∗∗	−0.132∗∗∗	0.020∗∗∗
	(0.008)	(0.009)	(0.003)
*N*	4134	4134	3723
*R*^*2*^	0.714	0.088	0.028
*Prov_FE*	Yes	Yes	Yes
*Year_FE*	Yes	Yes	Yes

Standard errors in parentheses, ∗*p* < 0.1, ∗∗*p* < 0.05, ∗∗∗*p* < 0.01.

#### 6.2.2 Cluster standard error tests

To further evaluate the robustness of the results, this paper incorporates a standard error test for clustering at the province level. As shown in Table 12, the results of the adjusted regression analysis indicate that the influence of postal and telecommunication service revenue on carbon emissions and carbon inequality remains significant. This suggests that the role of digital industrialization in these areas remains robust after controlling for intra-province autocorrelation. This finding further validates the reliability of the baseline conclusions, indicating that postal and telecommunication service revenue, as a proxy variable for digital industrialization, exert a significant impact on carbon emission-related indicators.

**Table 12:** Cluster standard error regression results.

	(1)	(2)	(3)
	*ln_carbon*	*lncar_inten*	*gini*
*lnpostal_telecom_revenue*	0.126∗∗∗	−0.014	−0.029∗∗∗
	(0.034)	(0.023)	(0.008)
*lngdp_per_capita*	0.426∗∗∗	0.234∗∗∗	0.000
	(0.055)	(0.065)	(0.015)
*lnpop*	0.526∗∗∗	0.075	0.026
	(0.068)	(0.048)	(0.016)
*lnscience*	0.101∗∗∗	−0.137∗∗∗	0.018∗∗
	(0.030)	(0.026)	(0.009)
*cons*	−6.943∗∗∗	3.607∗∗∗	0.218
	(0.528)	(0.745)	(0.160)
*N*	4149	4149	3738
*R*^*2*^	0.870	0.800	0.209
*Prov_FE*	Yes	Yes	Yes
*Year_FE*	Yes	Yes	Yes

Standard errors in parentheses, ∗*p* < 0.1, ∗∗*p* < 0.05, ∗∗∗*p* < 0.01.

#### 6.2.3 Balanced panel regression

To further validate the robustness of the findings, this study conducts a balanced panel regression analysis and controls for province and year fixed effects. As demonstrated in Table 13, the findings of the analysis indicate that the impacts of postal and telecommunications services revenue on carbon emissions and carbon inequality remain significant. The robustness test results provide further support the findings of the benchmark regression.

**Table 13:** Balanced panel regression results.

	(1)	(2)	(3)
	*ln_carbon*	*lncar_inten*	*gini*
*lnpostal_telecom_revenue*	0.169∗∗∗	0.025∗∗	−0.025∗∗∗
	(0.011)	(0.012)	(0.004)
*lngdp_per_capita*	0.369∗∗∗	0.191∗∗∗	0.002
	(0.017)	(0.019)	(0.007)
*lnpop*	0.473∗∗∗	0.010	0.020∗∗∗
	(0.017)	(0.018)	(0.006)
*lnscience*	0.109∗∗∗	−0.105∗∗∗	0.014∗∗∗
	(0.009)	(0.010)	(0.004)
*cons*	−6.661∗∗∗	3.676∗∗∗	0.239∗∗∗
	(0.166)	(0.178)	(0.061)
*N*	2863	2863	2590
*R*^*2*^	0.859	0.810	0.202
*Prov_FE*	Yes	Yes	Yes
*Year_FE*	Yes	Yes	Yes

Standard errors in parentheses, ∗*p* < 0.1, ∗∗*p* < 0.05, ∗∗∗*p* < 0.01.

#### 6.2.4 High-dimensional fixed effects model

To further ensure the robustness of the baseline results, this study employs high-dimensional fixed effects regression (HDFER), which specifically includes city-year high-dimensional fixed effects. The incorporation of city and year interaction fixed effects enables the model to account for the unobservables inherent to each city in distinct years, thereby enhancing its capacity to address potential heterogeneity and omitted variable issues.

Following the introduction of city-year high-dimensional fixed effects, the results are presented in Table 14. These findings indicate that the impact of postal and telecommunications services revenue on total carbon emissions and carbon inequality remain significant. The robustness test results provide further support the findings of the benchmark regression.

**Table 14:** High-dimensional fixed effects regression results.

	(1)	(2)	(3)
	ln_carbon	lncar_inten	gini
*lnpostal_telecom_revenue*	0.114∗∗∗	−0.018	−0.032∗∗∗
	(0.010)	(0.011)	(0.004)
*lngdp_per_capita*	0.422∗∗∗	0.268∗∗∗	−0.000
	(0.015)	(0.017)	(0.006)
*lnpop*	0.525∗∗∗	0.093∗∗∗	0.026∗∗∗
	(0.014)	(0.016)	(0.006)
*lnscience*	0.125∗∗∗	−0.149∗∗∗	0.023∗∗∗
	(0.008)	(0.009)	(0.003)
*cons*	−6.976∗∗∗	3.317∗∗∗	0.229∗∗∗
	(0.142)	(0.159)	(0.053)
*N*	4074	4074	3667
*R*^*2*^	0.871	0.809	0.221
*Prov_FE*	Yes	Yes	Yes
*Year_FE*	Yes	Yes	Yes
*City_Year_FE*	Yes	Yes	Yes

Standard errors in parentheses, ∗*p* < 0.1, ∗∗*p* < 0.05, ∗∗∗*p* < 0.01.

## 7 Mechanism analysis

### 7.1 Industrial digitization

In the mechanism analysis, the energy consumption intensity and enterprise net profit are selected as mediation variables for the industrial digitization. The energy consumption intensity is the ratio of energy consumption to production output, which reflects the efficiency of utilizing energy resources in industries. According to Ben Lahouel et al. [81] and Wang et al. [82], the industrial digitization can improve the energy efficiency and reduce the energy consumption intensity of corporations. Many traditional industries may experience digital advances to reduce energy consumption intensity, thereby affecting their carbon emissions and carbon inequality. Regarding the enterprise net profit, Industrial digitization tends to enhance the operational efficiency and competitiveness of firms, which in turn affects their profitability. Net profit may serve as an indicator of the economic advantages associated with industrial digitization. Alterations in these benefits may subsequently impact firms' investments and strategies in carbon emissions management [83,84]. Firms with greater profitability may possess the necessary resources to invest in green technologies, reduce carbon emissions, or enhance carbon efficiency. Concurrently, highly profitable firms may possess greater capacity to bear the costs of carbon emissions (e.g., carbon taxes or fees in an emissions trading system), thereby further mitigating inequalities in carbon emissions [85].Therefore, the enterprise net profit can also serve as an appropriate mediation variable.

The first stage regression results are shown in Table 15. Columns (1) and (2) show that the effect of postal and telecommunications service expenditures on energy consumption intensity is significantly negative, while the effect of postal and telecommunications service expenditures on net profit is significantly positive. These findings suggest that postal and telecommunications service expenditures are contribute to the improvement energy use efficiency within enterprises and increase net profits.

**Table 15:** The first stage mediation effect regression.

	(1)	(2)
	*lnenergy_y*	*net_profit*
*ln_postal_telecom_expen*	−0.157∗∗∗	0.106∗∗∗
	(0.008)	(0.008)
*ln_total_asset*	−0.233∗∗∗	0.950∗∗∗
	(0.009)	(0.009)
*ln_fuel_consum*	0.449∗∗∗	0.015∗∗∗
	(0.005)	(0.005)
*debt_ratio*	−0.497∗∗∗	−1.095∗∗∗
	(0.057)	(0.060)
*ln_inner_inno*	0.003	0.070∗∗∗
	(0.004)	(0.004)
*cons*	−2.859∗∗∗	−16.777∗∗∗
	(0.085)	(0.089)
*N*	25787	16509
*R*^*2*^	0.413	0.648
*Industry_FE*	Yes	Yes
*Year_FE*	Yes	Yes

Standard errors in parentheses, ∗*p* < 0.1, ∗∗*p* < 0.05, ∗∗∗*p* < 0.01.

The second stage regression results for the influence path of energy consumption intensity are shown in Table 16, and its effects on four dependent variables are significantly positive. After controlling the energy consumption intensity, the coefficients of postal and telecommunications service expenditures to total carbon emissions, carbon emission intensity and carbon inequality remain significant and increases, indicating the presence of partial mediation effect. Furthermore, the significant results of the Sobel test at the 1 % level also confirm the aforementioned mediation effects. These results demonstrate that the postal and telecommunications service expenditures can effectively reduce the carbon intensity and carbon inequality by reducing energy consumption intensity. Additionally, by reducing the energy consumption intensity, there is a consequential partial offset in the escalation of total carbon emissions caused by expenditures on postal and telecommunication services.

**Table 16:** The second stage mediation effect regression.

	(1)	(2)	(3)	(4)
	*log_carbon*	*log_emmi*	*log_eminten*	*gini*
*ln_postal_telecom_expen*	0.175∗∗∗	0.001	0.004∗∗∗	−0.018∗∗∗
	(0.007)	(0.001)	(0.001)	(0.001)
*lnenergy_y*	0.214∗∗∗	0.103∗∗∗	0.142∗∗∗	0.038∗∗∗
	(0.005)	(0.001)	(0.001)	(0.001)
*ln_total_asset*	0.214∗∗∗	−0.003∗∗	0.006∗∗∗	0.041∗∗∗
	(0.008)	(0.002)	(0.002)	(0.002)
*ln_fuel_consum*	0.249∗∗∗	−0.000	−0.014∗∗∗	−0.017∗∗∗
	(0.004)	(0.001)	(0.001)	(0.001)
*debt_ratio*	0.481∗∗∗	0.024∗∗	0.033∗∗∗	−0.050∗∗∗
	(0.047)	(0.010)	(0.010)	(0.010)
*ln_inner_inno*	−0.061∗∗∗	−0.007∗∗∗	−0.008∗∗∗	0.021∗∗∗
	(0.004)	(0.001)	(0.001)	(0.001)
*cons*	2.709∗∗∗	0.612∗∗∗	0.757∗∗∗	0.244∗∗∗
	(0.071)	(0.015)	(0.015)	(0.015)
*N*	25787	25787	25787	18206
*R*^*2*^	0.652	0.396	0.501	0.303
*Industry_FE*	Yes	Yes	Yes	Yes
*Year_FE*	Yes	Yes	Yes	Yes
*Sobel_Test*	−0.034∗∗∗	−0.16∗∗∗	−0.021∗∗∗	−0.007∗∗∗

Standard errors in parentheses, ∗*p* < 0.1, ∗∗*p* < 0.05, ∗∗∗*p* < 0.01.

The second stage regression results for the influence path of net profit are shown in Table 17. Its effects are significantly positive on total carbon emissions and negative on carbon intensity and carbon inequality. After controlling for net profits, the coefficients of postal and telecommunications service expenditures to total carbon emission, carbon intensity and carbon inequality remain significant, indicating the presence of partial mediation effects. The significant outcomes of Sobel test in Column (1)–(4) also confirm the aforementioned mediation effects. These inferential findings suggest that postal and telecommunications service expenditures increase total carbon emissions but decrease carbon intensity and carbon inequality through the channel of increasing net profits.

**Table 17:** The second stage mediation effect regression.

	(1)	(2)	(3)	(4)
	log_carbon	log_emmi	log_eminten	gini
*ln_postal_telecom_expen*	0.134∗∗∗	−0.011∗∗∗	−0.015∗∗∗	−0.025∗∗∗
	(0.008)	(0.002)	(0.002)	(0.002)
*ln_net_profit*	0.027∗∗∗	−0.006∗∗∗	−0.005∗∗	0.005∗∗∗
	(0.008)	(0.002)	(0.002)	(0.002)
*ln_total_asset*	0.140∗∗∗	−0.016∗∗∗	−0.019∗∗∗	0.029∗∗∗
	(0.012)	(0.003)	(0.003)	(0.003)
*ln_fuel_consum*	0.335∗∗∗	0.039∗∗∗	0.043∗∗∗	−0.000
	(0.005)	(0.001)	(0.001)	(0.001)
*debt_ratio*	0.427∗∗∗	−0.043∗∗∗	−0.046∗∗∗	−0.062∗∗∗
	(0.064)	(0.013)	(0.015)	(0.014)
*ln_inner_inno*	−0.060∗∗∗	−0.004∗∗∗	−0.005∗∗∗	0.020∗∗∗
	(0.004)	(0.001)	(0.001)	(0.001)
*cons*	2.544∗∗∗	0.164∗∗∗	0.224∗∗∗	0.215∗∗∗
	(0.166)	(0.035)	(0.040)	(0.036)
*N*	16509	16509	16509	11253
*R*^*2*^	0.625	0.162	0.157	0.258
*Industry_FE*	Yes	Yes	Yes	Yes
*Year_FE*	Yes	Yes	Yes	Yes
*Sobel_Test*	0.003∗∗∗	−0.001∗∗∗	−0.001∗∗	0.001∗∗∗

Standard errors in parentheses, ∗*p* < 0.1, ∗∗*p* < 0.05, ∗∗∗*p* < 0.01.

### 7.2 Digital industrialization

This research designates electricity energy consumption intensity at the city level as one of the mediator variables, represented by the ratio of total electricity consumption to GDP, as one of the mediator variables. Lin & Huang [86] finds that digital industrialization can optimize existing products, services, and infrastructure, and through the introduction of advanced information technology and data analytics, it can optimize energy use and reduce inefficient and excessive consumption. According to Zhang [58] and Hou et al. [87], electricity consumption volume may have negative effect on carbon intensity and carbon inequality. Therefore, electricity energy consumption intensity is theoretically considered as one suitable mediation variable. Furthermore, in line with prior studies [88,89], the progress of digital industrialization may have an influence on the industrial framework, leading to a decline in the manufacturing sector and a growth in the service sector. These transitions can impact a city's overall carbon emissions, and carbon inequality. Therefore, the industrial structure (the ratio of the tertiary industry to the secondary industry) is selected as the other mediation variable.

The first stage of the mediation effect regression is shown in Table 18, which specifically shows that the effects of postal and telecommunications services revenue and the ratio of postal and telecommunications revenue to GDP on electricity energy consumption intensity and industrial structure are both significantly positive at 1 % level. Collectively, these results indicate that both the increase in postal and telecommunications services revenue and ratio of postal and telecommunications revenue to GDP not only lead to higher energy consumption intensity, but also promote the optimization of the industrial structure.

**Table 18:** The first stage mediation effect regression.

	(1)	(2)
	*lnenergy_inten*	*industry_stru*
*lnpostal_telecom_revenue*	0.165∗∗∗	0.087∗∗∗
	(0.020)	(0.010)
*lngdp_per_capita*	0.279∗∗∗	−0.346∗∗∗
	(0.031)	(0.016)
*lnpop*	−0.485∗∗∗	−0.067∗∗∗
	(0.029)	(0.015)
*lnscience*	−0.062∗∗∗	0.097∗∗∗
	(0.017)	(0.009)
*cons*	−4.534∗∗∗	2.800∗∗∗
	(0.301)	(0.152)
*N*	4064	3928
*R*^*2*^	0.441	0.454
*Prov_FE*	Yes	Yes
*Year_FE*	Yes	Yes

Standard errors in parentheses, ∗*p* < 0.1, ∗∗*p* < 0.05, ∗∗∗*p* < 0.01.

The results of the second stage of mediation effect regression, with postal and telecommunications services revenue selected as the proxy variable, are depicted in Table 19, Table 20. Column (3) in Table 19 indicates that the effect of electricity energy consumption intensity on carbon inequality is significantly negative, while Column (1) indicates that there is no significant relationship between electricity energy consumption intensity and total carbon emissions. After controlling electricity energy consumption intensity, the coefficient between postal and telecommunications services revenue and carbon inequality remains significant and decreases in absolute value, indicating the existence of partial mediation effect. Columns (1) and (3) in Table 20 indicate that the effect of industrial structure on total carbon emissions and carbon inequality are significantly negative. After controlling industrial structure, the effects of postal and telecommunications services revenue on total carbon emission and carbon inequality remain significant, indicating the existence of partial mediation effect. Furthermore, the significant outcomes of Sobel test in Table 19, Table 20 confirm the aforementioned mediation effect.

**Table 19:** The second stage mediation effect regression 1.

	(1)	(2)	(3)
	*lncarbon*	*lnemission_inten*	*gini*
*lnpostal_telecom_revenue*	0.126∗∗∗	−0.023∗∗	−0.027∗∗∗
	(0.009)	(0.010)	(0.003)
*lnenergy_inten*	0.005	0.047∗∗∗	−0.008∗∗∗
	(0.007)	(0.008)	(0.003)
*lngdp_per_capita*	0.426∗∗∗	0.221∗∗∗	0.002
	(0.015)	(0.016)	(0.005)
*lnpop*	0.529∗∗∗	0.100∗∗∗	0.022∗∗∗
	(0.014)	(0.015)	(0.005)
*lnscience*	0.100∗∗∗	−0.134∗∗∗	0.018∗∗∗
	(0.008)	(0.009)	(0.003)
*cons*	−6.943∗∗∗	3.831∗∗∗	0.170∗∗∗
	(0.141)	(0.157)	(0.051)
*N*	4064	4064	3665
*R*^*2*^	0.870	0.803	0.212
*Prov_FE*	Yes	Yes	Yes
*Year_FE*	Yes	Yes	Yes
*Sobel_Test*	0.001	0.008∗∗∗	−0.002∗∗∗

Standard errors in parentheses, ∗*p* < 0.1, ∗∗*p* < 0.05, ∗∗∗*p* < 0.01, FE denotes fixed effects.

**Table 20:** The second stage mediation effect regression 2.

	(1)	(2)	(3)
	*lncarbon*	*lnemission_inten*	*gini*
*lnpostal_telecom_revenue*	0.133∗∗∗	0.003	−0.027∗∗∗
	(0.009)	(0.010)	(0.003)
*industry_stru*	−0.101∗∗∗	−0.199∗∗∗	−0.042∗∗∗
	(0.014)	(0.016)	(0.005)
*lngdp_per_capita*	0.387∗∗∗	0.160∗∗∗	−0.013∗∗
	(0.015)	(0.017)	(0.006)
*lnpop*	0.517∗∗∗	0.062∗∗∗	0.023∗∗∗
	(0.014)	(0.015)	(0.005)
*lnscience*	0.117∗∗∗	−0.116∗∗∗	0.023∗∗∗
	(0.008)	(0.009)	(0.003)
*cons*	−6.631∗∗∗	4.251∗∗∗	0.334∗∗∗
	(0.143)	(0.157)	(0.052)
*N*	3928.000	3928.000	3541.000
*R*^*2*^	0.873	0.801	0.226
*Prov_FE*	Yes	Yes	Yes
*Year_FE*	Yes	Yes	Yes
*Sobel_Test*	−0.009∗∗∗	−0.017∗∗∗	−0.003∗∗∗

Standard errors in parentheses, ∗*p* < 0.1, ∗∗*p* < 0.05, ∗∗∗*p* < 0.01.

In conclusion, postal and telecommunications services revenue impact total carbon emissions through industrial structure optimization only, while they impact carbon inequality by controlling electricity energy consumption intensity and promoting industrial structure optimization.

## 8 Conclusions and policy implications

### 8.1 Conclusions

Studies on the effects of digital economy on carbon emissions and carbon inequality have proliferated in recent years, but the existing literature still predominantly conceptualize the digital economy as a unified entity. Therefore, this paper divides the digital economy into two categories, industrial digitization and digital industrialization, to discuss the impact of the digital economy on carbon emissions and carbon inequality from two different perspectives. The industrial digitization data is the panel data of 82 industries in China from 2009 to 2015, collected from the China Enterprise Taxation Survey Data, while the digital industrialization data is the panel data of 301 cities in China from 2002 to 2017, obtained from the China City Statistical Yearbook and the China City Energy Statistical Yearbook. Utilizing these datasets, this paper employs a two-way fixed-effects model (TWFE) for empirical analysis and is conduct the mediation analysis from four perspectives: energy consumption intensity, enterprise technology purchase costs, electricity consumption intensity, and industrial structure. Our findings illuminate the intricate dynamics within China’s digital economy and its environmental impact. The main conclusions of our study are given as follows.

(1) The industrial digitization regressions show that, with a 1 % increase in postal and telecommunications service expenditures, total carbon emissions increase by 0.142 %, while carbon intensity (measured in two ways) and Gini index decrease by 0.011 %, 0.012 % and 0.041 respectively. This reflects the complexity and multidimensionality of industrial digitization development in term of environmental impacts. This result indicates that the direct and indirect consumption of energy required by the development of industrial digitization exacerbate environmental pressures and increase total carbon emissions. By improving production efficiency and optimizing resource allocation, industrial digitization has the potential to reduce the carbon intensity per unit of economic output and to promote a fair distribution of carbon emissions.
(2) The digital industrialization regressions show that, with a 1 % increase in postal and telecommunications service revenue and its proportion of GDP, total carbon emissions increase by 0.126 %, while Gini index decreases by 0.029. Similar to industrial digitization, the deepening of digital industrialization has also led to a higher intensity of energy demand, resulting in increased carbon emissions. This highlights the relationship between economic growth and environmental pressures. Digital industrialization facilitates information flow and optimizes resource allocation, which results in a more equitable distribution of carbon emissions.
(3) Industrial digitization can effectively reduce the carbon intensity and carbon inequality by reducing energy consumption intensity and increasing enterprise net profits. Additionally, by reducing the energy consumption intensity, there is a consequential partial offset in the escalation of total carbon emissions caused by industrial digitization.
(4) Digital industrialization impacts total carbon emissions through industrial structure optimization only, while they impact on carbon inequality controlling electricity energy consumption intensity and promoting industrial structure optimization. However, this paper does not find a significant relationship between digital industrialization and carbon emission intensity.

### 8.2 Policy suggestions

Based on the results above, we provide the following policy implications.

(1) In light of the observed rise in total carbon emissions resulting from industrial digitalization and digital industrialization, it is imperative that policymakers implement measures to regulate and mitigate the associated negative environmental impact. This research indicates a positive correlation between spending on postal and telecommunications services and total carbon emissions, which suggests that while digitalization drives economic growth, it also results in increased energy consumption. Consequently, policy initiatives should prioritize the promotion of renewable energy and energy-saving technologies in these sectors, with the objective of reducing reliance on fossil fuels. Concurrently, governments should reinforce energy efficiency in high-energy-consuming industries, through regulatory and incentive policies. This will encourage companies to adopt more environmentally friendly production methods and technologies. This will guarantee that the digitalization process does not result in an exponential increase in carbon emissions. The digital economy may continue to develop without increasing environmental burdens by introducing green energy and improving energy efficiency management.
(2) The advent of industrial digitalization offers a substantial opportunity to diminish carbon emission intensity and address the issue of carbon inequality. The research indicates that, while industrial digitalization may result in an overall increase in total carbon emissions, it has the effect of reducing carbon emission intensity and narrowing the gap in carbon emissions between different industries. This is achieved by enhancing production efficiency and optimizing the allocation of resources. It is therefore recommended that policymakers facilitate the wider adoption of digitalization across industries, with a particular focus on manufacturing and services. This may be achieved by encouraging the implementation of smart production and operational models. Governments may facilitate the advancement of digital technologies by augmenting financial resources allocated to research and by encouraging businesses to leverage digital methodologies to optimize production processes, reduce energy wastage, and diminish carbon emission intensity. Furthermore, it is imperative to reinforce regional policy coordination, with augmented investment in digital infrastructure in less developed regions, to guarantee that the carbon reduction benefits of digitalization are equitably distributed across diverse regions and industries. This will contribute to the achievement of the overall carbon reduction targets.
(3) The analysis of mechanisms presented in this paper indicates that although industrial digitalization increases carbon emissions, this growth can be partially mitigated by reducing energy consumption intensity and boosting corporate net profits. It is therefore recommended that policymakers intensify the promotion of energy-saving technologies, encouraging companies to utilize digital technologies to enhance energy efficiency and reduce reliance on traditional energy sources. Concurrently, the government should encourage companies to invest more resources in the research and application of green technologies by enhancing production efficiency and profitability through digitalization, thereby creating a positive feedback loop. The implementation of incentives, such as tax breaks and government subsidies, will serve to motivate companies to reduce their energy consumption intensity while simultaneously improving economic efficiency. This approach not only facilitates the achievement of carbon reduction objectives but also enhances corporate competitiveness, thereby propelling sustainable economic development.
(4) The optimization of industrial structure represents a pivotal strategy for the control of total carbon emissions and the reduction of carbon inequality in the context of digital industrialization. The research indicates that digital industrialization reduces carbon emission inequality primarily through the optimization of industrial structure, especially during the transition of high-energy-consuming sectors to low-carbon industries. It is recommended that policymakers prioritize the digital transformation of traditional high-energy-consuming industries, encouraging these sectors to adopt smart manufacturing, automation, and information technologies. This would result in a gradual reduction in their energy dependence and facilitate a transition towards low-carbon, high-value-added, technology-intensive industries. By providing policy support and financial investment, the government can facilitate the structural adjustments of these industries, thereby promoting a low-carbon transition in the industrial structure and reducing carbon emissions. Furthermore, it is recommended that the government facilitate collaboration and knowledge exchange between industries, with the aim of promoting the application of low-carbon technologies and management practices in high-carbon sectors. This would serve to further narrow the gap in carbon emissions between different industries, thereby achieving a more balanced and comprehensive strategy for reducing carbon emissions.

### 8.3 Further research

Our analysis has significantly contributed to the existing literature by elucidating the effects and pathways through which digital economy influences carbon emissions. Nonetheless, some limitations persist that ought to be addressed in subsequent research.

Firstly, due to the data limitations, this paper uses data up to 2017, but the rapid growth of the digital economy and its infrastructure suggests the need to include more recent data. Future studies should aim to incorporate data beyond 2017 to capture the latest trends and impacts of digitalization on carbon emissions. This will help to understand ongoing and emerging patterns in the context of industrial digitization and digital industrialization. Secondly, this paper focuses on China, providing valuable insights into the digital economy’s role in carbon emissions within a developing country context. However, the environmental impacts of digital economy may vary significantly across different geopolitical and economic contexts. Future research should aim to conduct comparative studies across various countries and regions, especially contrasting developed and developing economies, to uncover universal trends and country-specific characteristics.

## CRediT authorship contribution statement

**Yanze Wang:** Writing – original draft, Investigation, Data curation. **Zhi Su:** Writing – review & editing, Funding acquisition, Conceptualization. **Xuanye Cai:** Writing – review & editing, Methodology, Data curation. **Jian Yu:** Writing – review & editing, Conceptualization.

## Data availability statement

Data available on request from the authors.

## Declaration of competing interest

The authors declare that they have no known competing financial interests or personal relationships that could have appeared to influence the work reported in this paper.
